# DFBAlab: a fast and reliable MATLAB code for dynamic flux balance analysis

**DOI:** 10.1186/s12859-014-0409-8

**Published:** 2014-12-18

**Authors:** Jose A Gomez, Kai Höffner, Paul I Barton

**Affiliations:** Process Systems Engineering Laboratory, Massachusetts Institute of Technology, Cambridge, 02139 MA USA

**Keywords:** Dynamic flux balance analysis, Nonsmooth dynamic systems, Linear programming, Lexicographic optimization

## Abstract

**Background:**

Dynamic Flux Balance Analysis (DFBA) is a dynamic simulation framework for biochemical processes. DFBA can be performed using different approaches such as static optimization (SOA), dynamic optimization (DOA), and direct approaches (DA). Few existing simulators address the theoretical and practical challenges of nonunique exchange fluxes or infeasible linear programs (LPs). Both are common sources of failure and inefficiencies for these simulators.

**Results:**

DFBAlab, a MATLAB-based simulator that uses the LP feasibility problem to obtain an extended system and lexicographic optimization to yield unique exchange fluxes, is presented. DFBAlab is able to simulate complex dynamic cultures with multiple species rapidly and reliably, including differential-algebraic equation (DAE) systems. In addition, DFBAlab’s running time scales linearly with the number of species models. Three examples are presented where the performance of COBRA, DyMMM and DFBAlab are compared.

**Conclusions:**

Lexicographic optimization is used to determine unique exchange fluxes which are necessary for a well-defined dynamic system. DFBAlab does not fail during numerical integration due to infeasible LPs. The extended system obtained through the LP feasibility problem in DFBAlab provides a penalty function that can be used in optimization algorithms.

**Electronic supplementary material:**

The online version of this article (doi:10.1186/s12859-014-0409-8) contains supplementary material, which is available to authorized users.

## Background

The acceleration in the process of genome sequencing in recent years has increased the availability of genome-scale metabolic network reconstructions for a variety of species. These genome-based networks can be used within the framework of flux balance analysis (FBA) to predict steady-state growth and uptake rates accurately [1]. Dynamic flux balance analysis (DFBA) enables the simulation of dynamic biological systems by assuming organisms reach steady state rapidly in response to changes in the extracellular environment. Then, the rates predicted by FBA are used to update the extracellular environment. There exist three approaches to simulate DFBA models: the static optimization approach (SOA) [2], the dynamic optimization approach [2] (DOA), and the direct approach (DA). The static optimization approach uses the Euler forward method, solving the embedded LPs at each time step. Since most DFBA models are stiff, small time steps are required for stability, making this approach computationally expensive. Meanwhile, the DOA approach discretizes the time horizon and optimizes simultaneously over the entire time period of interest by solving a nonlinear programming problem (NLP). The dimension of this NLP increases with time discretization, therefore it is limited to small-scale metabolic models [3]. Finally, a DA has been proposed recently by including the LP solver in the right-hand side evaluator for the ordinary differential equations (ODEs) and taking advantage of reliable implicit ODE integrators with adaptive step size for error control. At present, the DOA is rarely used due to the intractability of the resulting NLP. DFBA can be easily performed on MATLAB using the constraint-based reconstruction and analysis (COBRA) toolbox [1,4], which implements the SOA. Recently, the DA has been implemented by Hanly and Henson [5], Mao and Verwoerd in the ORCA toolbox [6], Zhuang *et al.* in the dynamic multispecies metabolic modeling (DyMMM) framework [7,8], and others. A comprehensive list of DFBA implementations can be found in Table I of [3]. COBRA, DyMMM and ORCA codes are available on the Web. Of these, only DyMMM allows community simulations. Since ORCA and DyMMM are extremely similar, only COBRA and DyMMM were implemented in the case studies presented.

These implementations present several shortcomings. The COBRA Toolbox uses a fixed time step and does not take advantage of the high quality built-in integrators provided by MATLAB. Simulation stability and accuracy are closely linked to a uniformly small step size which can greatly increase simulation time. It can fail if the extracellular conditions are close to the FBA model becoming infeasible. In addition, it uses a simple exchange flux bounding scheme that does not allow the implementation of Michaelis-Menten kinetics or other more complex dynamic behaviors such as day/night shifts for photosynthetic organisms or system feed and discharge rates. It does not allow community simulations.

The ORCA toolbox and the DyMMM framework use the MATLAB built-in integrators. ORCA simulates monocultures only, whereas DyMMM can simulate cocultures. The ORCA toolbox allows the implementation of Michaelis-Menten and Hill kinetics only, whereas DyMMM provides the flexibility to implement more complex dynamics such as day/night shifts for photosynthetic organisms or system feed and discharge rates. Both attempt to carry on with simulations when the FBA model is infeasible by setting the growth rate and exchange fluxes equal to zero and displaying a death phase message. This message may be displayed even though the system is not infeasible.

None of these implementations (COBRA, ORCA, and DyMMM) account for the solution of a linear program (LP) being a nonsingleton set. Therefore, exchange fluxes are not necessarily unique and the dynamic system is not well-defined. Nonunique optimal fluxes have been discussed elsewhere in [9] and [5]. If no effort is made to obtain unique fluxes, different integrators could yield different results.

Höffner *et al.* have designed a fast and reliable community simulator that has the flexibility of implementing complex dynamics, does not fail, identifies precisely when a system becomes infeasible, and performs lexicographic optimization to render unique exchange fluxes [3]. In particular, it avoids numerical failure by reformulating the LP as an algebraic system and integrating an index-1 differential-algebraic equation (DAE) system. Despite these advantages, this simulator has not been widely used due to being coded in FORTRAN. In this paper, we implement the LP feasibility problem combined with lexicographic optimization in our Dynamic Flux Balance Analysis laboratory (DFBAlab), a MATLAB code that performs fast, reliable and flexible community simulations.

## Implementation

DFBAlab provides a solution to two major difficulties in existing implementations: nonunique exchange fluxes in the solution vector of an LP and the LP becoming infeasible when evaluating the ODE right-hand side close to the boundary of feasibility. DFBAlab implements lexicographic optimization to obtain unique exchange fluxes [3] and uses the LP feasibility problem to avoid obtaining infeasible LPs while running the simulation. DFBAlab runs using the commercial linear program solvers CPLEX [10], Gurobi [11], and MOSEK [12] and is compatible with the COBRA toolbox model format.

### Lexicographic optimization

Dynamic flux balance analysis is defined in the following way. Consider a vector **x**_0_ containing the initial concentrations of metabolites and biomass in a culture and assume there are *n*_*s*_ microbial species in the culture. Given some uptake and production rates of metabolites for each species (exchange fluxes), feed and discharge rates from the culture, mass transfer rates, and other dynamic processes, a rate of change function **f** can be obtained for each of the components of **x**_0_. The function **f** can then be integrated to find the concentration profiles with respect to time, **x**(*t*). Each species has a metabolic network represented by a stoichiometry matrix $\mathbf {S}^{k} \in \mathbb {R}^{{n_{q}^{k}} \times {n_{r}^{k}}}$ where ${n_{q}^{k}}$ are the number of metabolites and ${n_{r}^{k}}$ are the number of reactions in the metabolic network of species *k*. Consider that each species *k* has ${n_{h}^{k}}$ exchange fluxes. Then,

(1) $$ \begin{aligned} \dot{\mathbf{x}}(t) &= \mathbf{f}\left(t,\mathbf{h}^{1}(\mathbf{x}(t)),\hdots,\mathbf{h}^{n_{s}}(\mathbf{x}(t))\right), \; \forall t \in (t_{0}, t_{f}], \\ \mathbf{x}(t_{0}) &= \mathbf{x}_{0}, \end{aligned}  $$

where **h**^*k*^ is a vector containing the exchange fluxes of species *k* and is obtained by solving a linear program:

(2) $$ \begin{aligned} \underset{\mathbf{v}\in \mathbb{R}^{n_{r}^{k}}}{\max} \; (\mathbf{c}^k)^{\mathrm{T}} \mathbf{v}, \; \\ \text{s.t. } \textbf{S}^k&\textbf{v} = \mathbf{0}, \\ &\mathbf{v}^{k}_{UB}(\mathbf{x}(t)) \geq \mathbf{v} \ge \mathbf{v}^{k}_{LB}(\mathbf{x}(t)), \end{aligned}  $$

where $\mathbf {c}^{k} \in \mathbb {R}^{{n_{r}^{k}}}$ is the cost vector that maximizes growth fluxes, and $\mathbf {v}^{k}_{\textit {LB}}, \mathbf {v}^{k}_{\textit {UB}}$ are lower and upper bounds as functions of the extracellular concentrations. The vector **h**^*k*^ then takes the solution of this linear program to find the values of the exchange fluxes (*e.g.* biomass production rate, O _2_ consumption rate, ethanol production rate, etc.). This definition of DFBA has a serious problem: the solution set of the LP (2) can be nonunique (*e.g.* different flux distributions **v** can attain the maximum growth rate) and it is not clear which flux distribution should **h**^*k*^ take to carry-on with the integration.

Höffner and coworkers [3] use lexicographic optimization to render unique exchange fluxes. Lexicographic optimization works in the following way. First, it orders a number of objectives in a priority list. The highest priority objective is optimized first; then its optimum value is added as a constraint and the next objective in priority is optimized, and so on. Lexicographic optimization can be implemented in DFBA systems: the first objective is maximization of biomass; then all other exchange fluxes that appear in the right-hand side of (1) are added to the priority list. Note that the choice of the objective functions and their ordering are part of the model description and must be provided by the user. Although LPs don’t necessarily have a unique flux distribution that attains the optimal objective function value, they do have a unique optimal objective function value. This optimal objective function value changes continuously with changes in $\mathbf {v}^{k}_{\textit {LB}}, \mathbf {v}^{k}_{\textit {UB}}$. By making all the exchange fluxes that appear in the right-hand side of (1) optimization objectives ordered by priority, unique exchange fluxes are obtained, these exchange fluxes change continuously with respect to time and the integrator is able to carry-on integration reliably. Additional file 1 presents all the mathematical details pertaining to lexicographic optimization.

Harwood *et al.* (Harwood, S.M., Höffner, K. and Barton, P.I.: Solution of ordinary differential equations with a linear program embedded: the right-hand side case, Submitted) present an efficient algorithm to compute a basis that contains optimal bases for all LPs in the priority list. This algorithm was not implemented in DFBAlab because of difficulties in extracting the optimal basis information with no artificial variables from LP solvers in MATLAB, but will be implemented in future releases.

### LP feasibility problem

A major problem for DFBA simulators is that the LP in (2) may become infeasible as time progresses. There are two situations where the LP may become infeasible:

1. The problem is truly infeasible and the solution cannot be continued: in this case the integration should be terminated.
2. The problem is not infeasible but the LP becomes infeasible while the numerical integrator performs various operations to take a time step in (1): in this case the DFBA simulator in COBRA may fail to continue the simulation and ORCA and DyMMM will erroneously display death phase messages. In particular, the MATLAB’s built-in integrators will have a hard-time obtaining reliable right-hand side information as the system changes abruptly from being defined by the solution to (2), to being defined by an artificial solution that sets growth rates and exchange fluxes equal to zero.

In this paper we use the LP feasibility problem [13] combined with lexicographic optimization to generate an extended dynamic system for which the LP always has a solution. An LP feasibility problem finds a feasible point or identifies an LP as infeasible. It has two main characteristics: it is always feasible and its optimal objective function value is zero if and only if the original LP is feasible. Several different versions of the LP feasibility problem can be constructed by adding some slack variables to the constraints. For the LP formulation in (2), the following is an LP feasibility problem:

(3) $$ \begin{aligned} \underset{\begin{subarray}{c} \mathbf{v}\in \mathbb{R}^{n_{r}^{k}}, \\ \mathbf{s}_+, \mathbf{s}_- \in \mathbb{R}^{n_{q}^{k}} \end{subarray}}{\min} \; \; \; \sum \limits_{i=1}^{n_{q}^{k}} s_{+i} &+ s_{-i}, \\ \text{s.t. } \textbf{S}^k&\textbf{v} + \mathbf{s}_+ - \mathbf{s}_-= \mathbf{0}, \\ &\mathbf{v}^{k}_{UB}(\mathbf{x}(t)) \geq \mathbf{v} \ge \mathbf{v}^{k}_{LB}(\mathbf{x}(t)), \\ &\mathbf{s}_+ \geq \mathbf{0}, \; \mathbf{s}_- \geq \mathbf{0}. \end{aligned}  $$

Let **S**_*i*_ be the *i*^*th*^ row of **S**. When an LP is constructed in this form, a feasible solution is obtained by finding a **v** such that $\mathbf {v}^{k}_{\textit {UB}}(\mathbf {x}(t)) \geq \mathbf {v} \ge \mathbf {v}^{k}_{\textit {LB}}(\mathbf {x}(t))$ and then letting $s_{+i} = -{\mathbf {S}^{k}_{i}} \mathbf {v}$ and *s*_−*i*_=0 if ${\mathbf {S}^{k}_{i}}\mathbf {v}<0$, or $s_{-i} = {\mathbf {S}^{k}_{i}} \mathbf {v}$ and *s*_+*i*_=0 otherwise. DFBAlab transforms LP (2) to standard form and then obtains the LP feasibility problem for an LP in standard form [13]; however, the principles are the same. A detailed explanation can be seen in the Additional file 1.

DFBAlab uses the LP feasibility problem (3) instead of (2) to find the growth rates and exchange fluxes for each species in the culture. It sets the feasibility cost vector as the top priority objective in the lexicographic optimization scheme. Then, the second-priority linear program maximizes biomass and the subsequent lower-priority LPs obtain unique exchange fluxes. The order of the exchange fluxes in the priority list is user-defined. The priority list order is fixed throughout the simulation. This order has to be defined carefully or unrealistic simulation results may be obtained (as illustrated in Example 2). This approach has the following advantages:

1. The dynamic system in (1) is defined for all simulation time.
2. The integrator does not encounter infeasible LPs while taking a step and is able to obtain reliable right-hand side information speeding up the integration process.
3. The objective function value of (3) provides a distance from feasibility and can be integrated providing a penalty function that can be useful for optimization purposes. Only trajectories with penalty function value equal to zero (within some tolerance *ε*) are feasible.

## Results and discussion

The following examples demonstrate the reliability and speed of DFBAlab compared to existing implementations of the SOA and DA. SOA is represented by the COBRA dFBA implementation and DA by the DyMMM implementation. In the first example, a monoculture of *E. coli* is simulated with all three methods. In the second example, a coculture of algae and yeast is simulated using DFBAlab and DyMMM. In the third example, this same coculture is simulated considering the pH balance. Finally, the last example shows how DFBAlab running time increases linearly with the number of FBA models in the system. All running times are for a 3.20 GHz Intel®; Xeon®; CPU in MATLAB 7.12 (R2011a), Windows 7 64-bit operating system using LP solver CPLEX. All running times are for the integration process only (preprocessing times are not reported). DFBA models are usually stiff; therefore, ode15s, MATLAB’s integrator for stiff systems, was used for all simulations.

### Example 1

**Figure 1:**
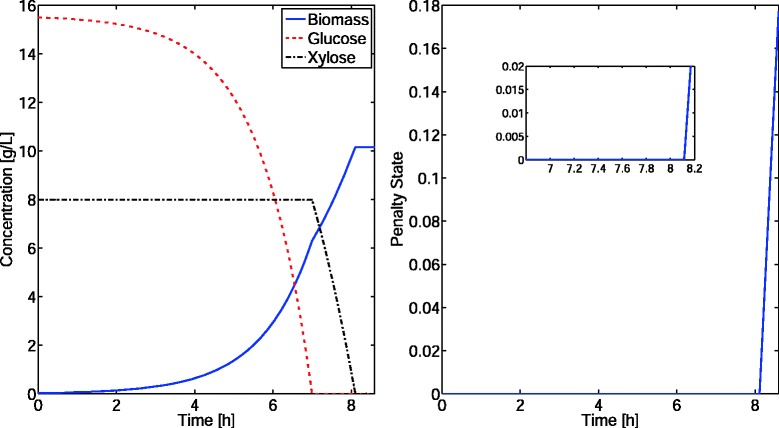
**Concentration profiles (left) and DFBAlab penalty function (right) of Example 1.** The penalty function shows how the simulation becomes infeasible after approximately 8.1 hours. Simulation times: DyMMM = 6.6 seconds, DFBAlab = 7.7 seconds.

This is Example 6.2 in (Harwood, S.M., Höffner, K. and Barton, P.I.: Solution of ordinary differential equations with a linear program embedded: the right-hand side case, Submitted) which is based on [5]. Here we compare the performance of COBRA, DyMMM and DFBAlab simulating an *E. coli* monoculture. The metabolic network reconstruction used was iJR904 published in [14]. This metabolic model contains 2191 reactions and 1706 metabolites. Initial conditions were 0.03 g/L of inoculum, 15.5 g/L of glucose and 8 g/L of xylose. Oxygen concentration was kept constant at 0.24 mmol/L. Michaelis-Menten expressions with inhibition terms were implemented to bound the uptake of glucose, xylose and oxygen using the parameters presented in Table I and Equations (3), (4) and (5) in [5]. DFBAlab obtained unique fluxes by minimizing ethanol production, and then glucose and xylose consumption, after maximizing biomass, using lexicographic optimization. The COBRA simulator performed poorly. Since COBRA does not have the flexibility to implement Michaelis-Menten expressions, the simulation results were incorrect. In addition, the fixed step size slowed down the integration process. Non-negativity constraints for all states variables were enforced in both DyMMM and DFBAlab, by using the ‘Nonnegative’ option. DyMMM and DFBAlab obtained the same concentration profiles presented in Figure 1. DyMMM has a good performance recovering from a frequent failure point occurring when growth switches from glucose-based to xylose-based. DFBAlab performs a little bit slower than DyMMM due to the four additional LPs being solved to perform lexicographic optimization, obtaining at least the same level of accuracy. Finally, the penalty function indicates that the system becomes infeasible after approximately 8.1 hours (Figure 1).

### Example 2

This is an example from [15] of a coculture of the microalgae *Chlamydomonas reinhardtii* and *Saccharomyces cerevisiae* (yeast) in a continuous stirred-tank reactor (CSTR) reactor. The genome-scale metabolic network reconstructions used were iRC1080, comprising 2191 reactions and 1706 metabolites from [16], and iND750, comprising 1266 reactions and 1061 metabolites from [17], for algae and yeast, respectively. In this simulation, yeast consumes glucose to produce CO_2_ while algae consumes mainly CO_2_ to produce O_2_ during the day, and acetate to produce CO_2_ during the night. The dynamic mass balance equations of the extracellular environment for this system are:

(4) $$\begin{array}{@{}rcl@{}} \dot y^{i}(t) &=& \mu^{i} \left(\mathbf{x}(t)\right) y^{i}(t) - \frac{F_{out}y^{i}(t)}{V}, \end{array} $$

(5) $$\begin{array}{@{}rcl@{}} \dot s(t) &=& \frac{F_{in}s_{0} - F_{out}s(t)}{V} + MT_{s}(\mathbf{x}(t)) \\ &&+ \sum \limits_{i} \left(v_{s_{p}}^{i}\left(\mathbf{x}(t)\right)-v_{s_{c}}^{i} \left(\mathbf{x}(t)\right)\right)y^{i}(t), \\ \text{for } i &=& Y, A,\, \text{and for}\ s = g, o, c, e, a, \notag \end{array} $$

where *y*^*i*^, *g*, *o*, *c*, *e*, and *a* correspond to the concentrations of biomass of species *i*, glucose, oxygen, carbon dioxide, ethanol and acetate, respectively. The superscripts *Y*,*A* refer to yeast and algae, **x**= [*y*^*Y*^*y*^*A*^*gocea*], *μ*^*i*^ is the growth rate of species *i*, $v_{s_{c}}^{i}$ and $v_{s_{p}}^{i}$ are the consumption and production rates of substrate *s* for species *i* determined through lexicographic optimization, *s*_0_ is the concentration of *s* in the feed, *F*_*in*_ and *F*_*out*_ are the inlet and outlet flows, *V* is the volume of the system, and *MT*_*s*_ is the mass transfer rate of *s* given by the following expression:

(6) $$ \begin{aligned} MT_{s}(\mathbf{x}(t)) = \left\{ \begin{array}{ll} (k_{sL}\theta)\left(\frac{s^{(g)}}{K_{Hs}}-s(t) \right) & \quad \text{for } s = o, c, \\ 0 & \quad \text{for } s = g, e, a, \end{array} \right. \end{aligned}  $$

where *K*_*Hs*_ refers to Henry’s constant of component *s* at 25°C, *k*_*sL*_*θ* is the mass transfer coefficient for component *s* obtained from [18], and *s*^(*g*)^ is the concentration of *s* in the atmosphere. The maximum concentration of oxygen and carbon dioxide in the culture is bounded by Henry’s constant:

(7) $$ s((t)) \leq K_{Hs}, \; \forall t \in \left[t_{0}, t_{f}\right] \; \text{for}\ s = o, c.  $$

Initial concentrations and other parameters are presented in Table 1. The uptake kinetics are bounded above by the Michaelis-Menten expression:

(8) $$ v_{s}^{i,UB}(s(t))=v_{s,max}^{i}\frac{s(t)}{{K_{s}^{i}}+s(t)},  $$

**Table 1:** **Initial concentrations and parameters of example 2**

	**Variable**	**Simulation 1**	**Simulation 2**		**Parameters**	
	$y^{\text {Y}}_{0}$	1.10	0.71	gDW/L	*V*_0_	140 L
	$y^{\text {A}}_{0}$	1.86	1.80	gDW/L	*F*_*in*_	1 L/h
	*g*_0_	1.40 E^−2^	2.28 E^−2^	mmol/L	*F*_*out*_	1 L/h
	*o*_0_	6.53 E^−4^	5.57 E^−4^	mmol/L		
	*c*_0_	1.06	1.03	mmol/L		
	*e*_0_	8.21	17.32	mmol/L		
	*a*_0_	2.39 E^−2^	2.48 E^−2^	mmol/L		

Simulation 1 used the priority list presented in Table 2, while for Simulation 2 objective 4 for algae was inverted.

**Table 2:** **Priority list order for the lexicographic linear programs in example 2**

	**Yeast**	**Algae**
1	Minimize slacks of	Minimize slacks of
	feasibility LP	feasibility LP
2	Maximize biomass	Maximize biomass
	production	production
3	Minimize glucose	Maximize acetate
	consumption	consumption
4	Minimize O_2_	Minimize O_2_
	consumption	consumption and maximize
		O _2_ production
5	Maximize CO_2_ production	Maximize CO_2_ consumption
6	Maximize ethanol production	

for *i*=*Y*,*A* and *s*=*a*,*o*,*c* with $v_{s,max}^{i}$ and ${K_{s}^{i}}$ obtained from [19], [20] and [21] for acetate, carbon dioxide and oxygen. Production of oxygen by algae, ethanol by yeast, and carbon dioxide by algae and yeast were not bounded.

In addition to the extracellular concentrations, algae growth is affected by light availability because it is a photosynthetic organism. Day and night shifts were simulated using the following surface light function:

(9) $$ \begin{aligned} I_{0}(t) &= 28 \frac{\max \left (\sin^{2} \left (\frac{2\pi t}{48}\right),\sin^{2} \left (\frac{10\pi}{48}\right) \right)-\sin^{2} \left (\frac{10\pi}{48}\right)}{1-\sin^{2} \left (\frac{10\pi}{48}\right)} \\ &\!(=) \; \frac{\text{mmol photons}}{gDW \times h}. \end{aligned}  $$

This light function simulates daylight from 5:00 to 19:00. The prefactor was obtained from [22]. The Beer-Lambert law was used to average the light available to algae cells considering that higher biomass densities block light and deeper sections of the pond receive less sunlight:

(10) $$ \begin{aligned} I_{a}(t,\mathbf{x}(t)) &= I_{0}(t) \frac{1-\exp \left(-L K_{e}(\mathbf{x}(t)) \right)}{L K_{e}(\mathbf{x}(t))}, \\ &\!(=) \; \frac{\text{mmol photons}}{gDW \times h}, \end{aligned}  $$

where *K*_*e*_(**x**(*t*)) is a linear function of the concentration of biomass in the culture and *L* is the pond depth [21]. Concentration variations of biomass for different pond depths were neglected.

**Figure 2:**
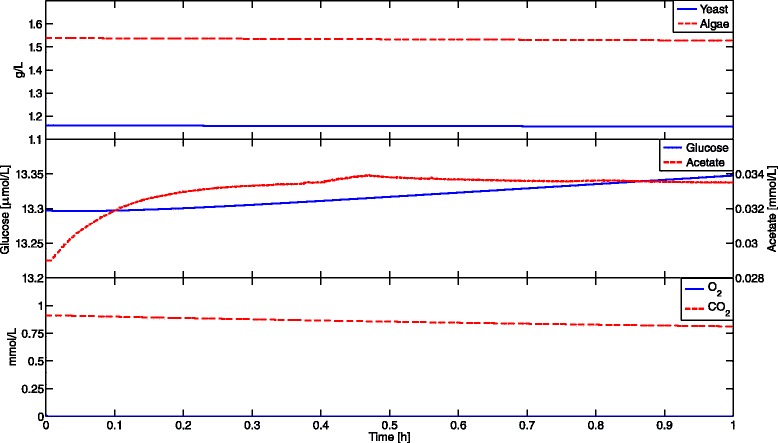
**DyMMM simulation results of example 2.** DyMMM is unable to simulate Example 2. Computation time for one hour of simulation was of more than 3900 seconds using MATLAB integrator ode45. In addition, the acetate curve has several points of nonsmoothness that can be explained by the presence of nonunique fluxes. Numerical integrators are unable to integrate these kinds of systems.

**Figure 3:**
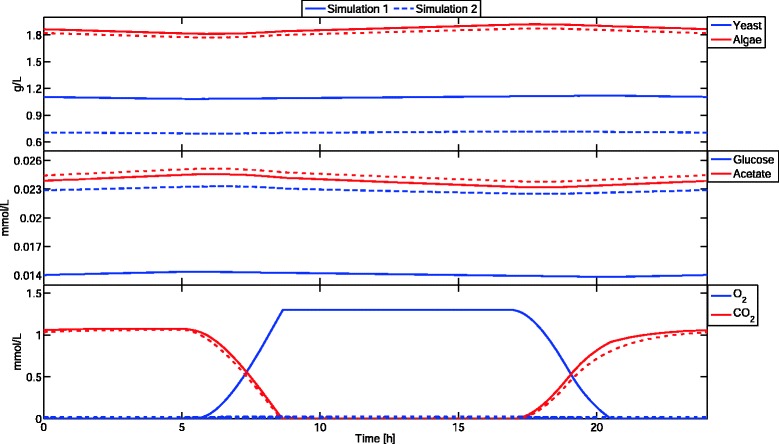
**DFBAlab simulation results of example 2.** Two cyclic steady states are presented. Simulation 1 (solid line) was performed with lexicographic objectives presented in Table 2, whereas simulation 2 (dashed line) used the negative of Objective 4 for algae. Significant differences can be observed in the predicted concentrations of yeast, glucose, and oxygen. Computation times for simulations 1 and 2 where 82 and 74 seconds, respectively.

This complex community simulation cannot be carried out using the DFBA simulator in COBRA. Non-negativity constraints were enforced for all state variables in both, DyMMM and DFBAlab, by using the ‘Nonnegative’ option. After more than 10,000 seconds of running time using MATLAB implicit integrator ode15s, the simulation on DyMMM was stopped. Using explicit integrator ode45 instead, DyMMM took more than 3900 seconds to simulate one hour of the cyclic steady-state of this coculture and the results are inaccurate. This is expected because explicit integrators can calculate new steps as long as they are able to evaluate the right-hand side of the ODE. The results obtained by DyMMM using ode45 are inaccurate because explicit integrators should not be used for stiff systems, and the right-hand side is nonunique. In Figure 2, it can be seen that the acetate curve presents several points of nonsmoothness which are expected in systems with nonunique fluxes. Numerical integrators are unable to handle these systems as they encounter discontinuous exchange fluxes when decreasing step-size. Therefore, computation time is excessive and the results are incorrect. This shortcoming is addressed by DFBAlab using six lexicographic optimizations for yeast and five for algae. It took only 82 seconds to simulate accurately 24 hours of this coculture using the lexicographic objectives shown in Table 2, and 74 seconds to simulate this same system with Objective 4 for algae inverted. Simulation results can be seen in Figure 3.

Lexicographic optimization is very important in this example; if the negative of Objective 4 for algae is used, oxygen, acetate and yeast concentration profiles vary significantly. In particular, notice the large difference in the O _2_ concentration profile between the two simulations. Since the O _2_ flux is nonunique, selecting different fluxes will lead to different trajectories. Without a rule on how to choose a flux from the optimal solution set, DyMMM can choose different elements of this set while cutting its time step, obtaining unreliable right-hand side information. Therefore, it is not surprising that the DyMMM simulator was unable to simulate this system.

It must be noted that in reality, this difference is not observed in nature. When Objective 4 for algae is inverted (maximizing O _2_ consumption and minimizing O _2_ production), the model is able to uptake unlimited H ^+^ ions from the environment and produce water until the O _2_ uptake bound is reached. This behavior will change the pH of the system and the overconsumption of O _2_ would be unsustainable. Increased modeling efforts can bound the uptake of other substrates such as nitrogen, phosphorus and iron and use pH dependent uptakes. In this context, a pH balance will be necessary. This balance is implemented in Example 3. Finally, biologically relevant lexicographic objectives must be selected because some objectives may lead to unrealistic systems as the one just presented.

### Example 3

This example illustrates the modeling flexibility DFBAlab provides. The growth rate of autotrophic microalgae such as *C. reinhardtii* is dependent on CO _2_ concentration. This concentration is affected by pH, as the following equilibrium reactions are present in the extracellular environment:

(11) $$ \begin{aligned} \text{NH}_{3} + \text{H}_{2}\text{O} &\rightleftharpoons \text{NH}_{4}^{+} + \text{OH}^{-} \\ \text{CO}_{2} + \text{H}_{2}\text{O} &\rightleftharpoons \text{H}^{+} + \text{HCO}_{3}^{-} \rightleftharpoons 2\text{H}^{+} + \text{CO}_{3}^{2-} \\ \text{H}_{2}\text{O} &\rightleftharpoons \text{H}^{+} + \text{OH}^{-} \end{aligned}  $$

Using the equilibrium constants presented in Table one in [21] and the equilibrium model in Equations (14a) and (14b) in [21], a pH balance was introduced to Example 2. The pH balance introduces algebraic equations that have to be satisfied at all times. This kind of system is called a Differential-Algebraic Equation system (DAE) where some variables are algebraic variables (their time derivative is not calculated explicitly) and others are differential variables. To our knowledge, no one has introduced the pH equilibrium equations in a DFBA simulation before. To add the pH balance to the system, total carbon and total nitrogen were added to the differential variables, CO _2_ concentration was transformed into an algebraic variable, and new algebraic variables for NH$_{4}^{+}$, NH _3_, HCO$_{3}^{-}$, CO$_{3}^{2-}$, and H ^+^ concentrations were introduced. Total nitrogen in the system was assumed to be constant at 0.1643 mmol/L, which is the concentration present in the Charles River in Cambridge [23], the effect of H ^+^ exchange by algae on pH was considered negligible, and ionic valency of the solution was assumed to be equal to zero.

**Figure 4:**
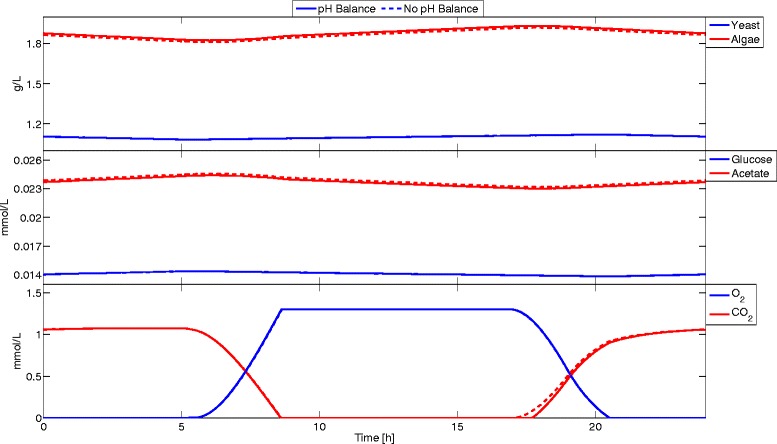
**DFBAlab simulation results of example 3.** This example incorporates the pH balance (solid line). Simulation results were close to the ones obtained without a pH balance. Slight variations were observed for the CO _2_ concentration profile. Computation time was 162 seconds.

**Figure 5:**
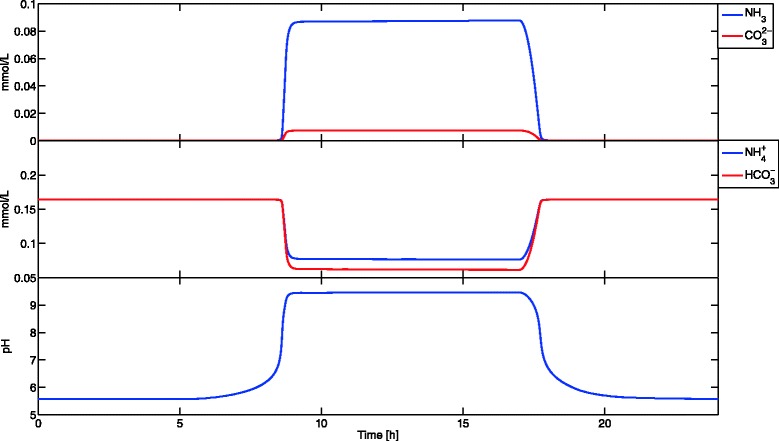
**Equilibrium species and pH of example 3.** The pH balance enables tracking of ionic concentration profiles. This information allows using pH dependent uptake kinetics and uptake kinetics for ionic species.

If the Jacobian of the algebraic equations with respect to the algebraic variables is nonsingular, the DAE is index-1 and can be solved with MATLAB ode15s. Table 3 shows the initial conditions and parameters used. No non-negativity constraints were enforced; however, the uptake kinetics were specified so that negative concentrations could not occur. Concentration profiles are presented in Figures 4 and 5. Simulation results with a pH balance are close to those without a pH balance. However, the information obtained from this simulation enables using pH dependent uptake kinetics and ionic species uptake kinetics leading to more accurate simulations. It took only 162 seconds to simulate accurately 24 hours of this coculture with a pH balance.

**Table 3:** **Initial concentrations and parameters of example 3**

	**Variable**	**No pH balance**	**pH balance**		**Parameters**	
	$y^{\text {Y}}_{0}$	1.10	1.10	gDW/L	*V*_0_	140 L
	$y^{\text {A}}_{0}$	1.86	1.87	gDW/L	*F*_*in*_	1 L/h
	*g*_0_	1.40 E^−2^	1.40 E^−2^	mmol/L	*F*_*out*_	1 L/h
	*o*_0_	6.53 E^−4^	6.52 E^−4^	mmol/L		
	*c*_0_	1.06	1.06	mmol/L		
	*e*_0_	8.21	8.21	mmol/L		
	*a*_0_	2.39 E^−2^	2.37 E^−2^	mmol/L		
	N _*T*_	-	1.64 E^−1^	mmol/L		
	C _*T*_	-	1.22	mmol/L		
	NH _3_	-	2.45 E^−5^	mmol/L		
	NH$_{4}^{+}$	-	1.64 E^−1^	mmol/L		
	HCO$_{3}^{-}$	-	1.64 E^−1^E^−2^	mmol/L		
	CO$_{3}^{2-}$	-	2.58 E^−6^	mmol/L		
	H ^+^	-	2.67 E^−6^	mmol/L		

### Example 4

In this example a monoculture of *Chlamydomonas reinhardtii* was simulated to illustrate how DFBAlab performs for simulations with a large number of species models. The parameters implemented in Example 2 were used with different initial conditions. No non-negativity constraints were enforced, but the uptake kinetics were specified so that negative concentrations could not happen. Algae biomass was split among several LPs and running times were compared. Table 4 shows the running times for 24 hours of simulation for different numbers of models in the system.

**Table 4:** **Running times of example 4 with increasing number of models**

**Number of models**	**Time (s)**
1	18.3
2	36.9
5	94.2
10	190
25	506

### Discussion

In these examples, the reliability and speed of DFBAlab has been shown compared to current open MATLAB benchmarks in DFBA simulation. COBRA lacks flexibility when implementing Michaelis-Menten kinetics and the use of a fixed time step decreases the accuracy of these simulations, or increases the integration time for very small time steps. DyMMM provides a flexible framework that allows the implementation of community simulations. However, if any of the exchange fluxes are nonunique, simulation results will be incorrect. DFBAlab uses lexicographic optimization to obtain a well-defined system, but it requires specification of lower-priority objective functions. Biologically relevant lower-priority objectives must be sought to restrict the solution set of (2) to a more realistic set. For instance, it has been suggested by a reviewer that maximization of ATP is a biologically relevant objective that should follow maximization of biomass. In DFBAlab, this objective can be added right after maximization of biomass. Then, the unique exchange fluxes obtained are guaranteed to maximize biomass first, and then maximize ATP. If other biologically relevant objectives are found, they can be added in the same way to the priority list (after maximization of biomass, but before the exchange fluxes), such that the exchange fluxes obtained are more realistic.

The DFBAlab framework is flexible enough to allow DAEs, which could result from performing pH balances in the culture. Furthermore, in community simulations, the running time of DFBAlab increases linearly with the number of species. The LP feasibility objective function in DFBAlab serves two purposes: it helps to distinguish between feasible and infeasible trajectories and it can serve as a penalty function in optimization algorithms. Future work will present the implementation of this penalty function in the context of DFBA optimization.

## Conclusions

The objective of this work is to provide an easy to use implementation that minimizes troubleshooting of numerical issues and facilitates focus on the analysis of simulation results. DFBAlab, a reliable DFBA simulator in MATLAB, is presented. DFBAlab uses lexicographic optimization to obtain unique exchange fluxes and a well-defined dynamic system. DFBAlab uses the LP feasibility problem to generate an extended dynamic system and a penalty function. DFBAlab performs better than its counterpart DyMMM in complex community simulations: it is faster and more accurate because the unique fluxes provided by lexicographic optimization are necessary for numerical integration. In addition, DFBAlab can integrate the DAEs resulting from implementing pH balances. Biologically relevant lower-priority objectives must be sought to perform lexicographic optimization. The penalty function provided by DFBAlab can be used to optimize DFBA systems. However, it should be noted that the FORTRAN code referred in [3] has advantages since it only takes about 30 seconds for Example 2 [15].

## Availability and requirements

The DFBAlab code is available, without charge, for both education and non-profit research purposes, at http://yoric.mit.edu/dfbalab.

**Project name:** DFBAlab

**Project homepage:**http://yoric.mit.edu/dfbalab

**Operating system(s):** Any operating system that supports MATLAB

**Programming language:** MATLAB’s programming language

**Other requirements:** An LP solver among CPLEX, Gurobi, or MOSEK

**License:** Terms of use need to be accepted before being able to download the code.

**Any restrictions to use by non-academics:** Not available for non-academics.

## Additional file

Additional file 1
**Detailed mathematical presentation of lexicographic optimization and the LP feasibility problem.**

## Abbreviations

COBRA: Constraint-based reconstruction and analysis
CSTR: Continous stirred-tank reactor
DA: Direct approach
DAE: Differential-algebraic equation
DFBA: Dynamic flux balance analysis
DFBAlab: Dynamic flux balance analysis laboratory
DOA: Dynamic optimization approach
DyMMM: Dynamic multispecies metabolic modeling
LP: Linear program
NLP: Nonlinear programming problem
ODE: Ordinary differential equation
SOA: Static optimization approach
