# Supplementary material for: DFBAlab: a fast and reliable MATLAB code for dynamic flux balance analysis
Source: BMC Bioinformatics. 2014 Dec 18;15(1):409. doi: 10.1186/s12859-014-0409-8 (PMC4279678; doi:10.1186/s12859-014-0409-8)
Supplement: Additional file 1 — Detailed mathematical presentation of lexicographic optimization and the LP feasibility problem. [file 12859_2014_409_MOESM1_ESM.pdf]

SOFTWARE

DFBAlab: A fast and reliable MATLAB code for Dynamic Flux Balance Analysis

Jose A Gomez, Kai Höffner and Paul I Barton*

*Correspondence: pib@mit.edu
Process Systems Engineering Laboratory, Massachusetts Institute of Technology, Cambridge, MA 02139
Full list of author information is available at the end of the article

Additional File #1

Lexicographic optimization

Dynamic flux balance analysis is defined in the following way. Consider a vector $\mathbf{x}_0$ containing the initial concentrations of metabolites and biomass in a culture and assume there are n_s microbial species in the culture. Given some uptake and production rates of metabolites for each species (exchange fluxes), feed and discharge rates from the culture, mass transfer rates, and other dynamic processes, a rate of change function $\mathbf{f}$ can be obtained for each of the components of $\mathbf{x}_0$. The function $\mathbf{f}$ can then be integrated to find the concentration profiles with respect to time, $\mathbf{x}(t)$. Consider that each species k has n_h^k exchange fluxes and define the linear maps $\mathbf{B}^k : \mathbb{R}^{n_r^k} \to \mathbb{R}^{n_h^k}$ which obtain the exchange fluxes from the n_r^k metabolic fluxes. Formally, given the nonempty open set $D_x \subset \mathbb{R}^{n_x}$, $\mathbf{f} : [t_0, t_f] \times D_x \times \mathbb{R}^{n_h^1} \times \ldots \times \mathbb{R}^{n_h^{n_s}} \to \mathbb{R}^{n_x}$, $\mathbf{v}^k : D_x \to \mathbb{R}^{n_r^k}$, $\mathbf{v}_{LB}^k : D_x \to \mathbb{R}^{n_r^k}$, $\mathbf{v}_{UB}^k : D_x \to \mathbb{R}^{n_r^k}$ for $k = 1, \ldots, n_s$, and $\mathbf{x} : [t_0, t_f] \to \mathbb{R}^{n_x}$:

$$\begin{aligned} \dot{\mathbf{x}}(t) &= \mathbf{f}(t, \mathbf{x}(t), \mathbf{B}^1(\mathbf{v}^1(\mathbf{x}(t))), \ldots, \mathbf{B}^{n_s}(\mathbf{v}^{n_s}(\mathbf{x}(t)))), \ \forall t \in (t_0, t_f], \\ \mathbf{x}(t_0) &= \mathbf{x}_0, \end{aligned} \tag{1}$$

where $\mathbf{v}^k$ is an element of the solution set of the flux balance model of species k:

$$\begin{aligned} \mathbf{v}^k(\mathbf{x}(t)) \in \arg \max_{\mathbf{v} \in \mathbb{R}^{n_r^k}} \ & (\mathbf{c}^k)^{\mathrm{T}} \mathbf{v}, \\ \text{s.t. } & \mathbf{S}^k \mathbf{v} = \mathbf{0}, \\ & \mathbf{v}_{UB}^k(\mathbf{x}(t)) \geq \mathbf{v} \geq \mathbf{v}_{LB}^k(\mathbf{x}(t)), \end{aligned} \tag{2}$$

where $\mathbf{S}^k \in \mathbb{R}^{n_q^k \times n_r^k}$ is the stoichiometry matrix, $\mathbf{c}^k \in \mathbb{R}^{n_r^k}$ is a vector of zeroes and ones with ones only in positions of growth fluxes, and $\mathbf{v}_{LB}^k, \mathbf{v}_{UB}^k$ are lower and upper bounds as functions of the extracellular concentrations. This definition of DFBA has a serious problem: the solution set of the LP (2) is a set-valued function, and therefore, when it is nonsingleton it is not clear which element of the set $\mathbf{v}^k$ should take to carry-on with the integration.

In the rest of this document, we will work with the *standard form* LP of (2). Let $\mathbf{A}^k \in \mathbb{R}^{n_m^k \times n_v^k}$, $\mathbf{c}^k \in \mathbb{R}^{n_v^k}$, $\boldsymbol{\beta} \in \mathbb{R}^{n_m^k}$:

$$\begin{aligned} \min_{\mathbf{v} \in \mathbb{R}^{n_v^k}} \ & (\mathbf{c}^k)^{\mathrm{T}} \mathbf{v}, \\ \text{s.t. } & \mathbf{A}^k \mathbf{v} = \boldsymbol{\beta}, \\ & \mathbf{v} \geq \mathbf{0}. \end{aligned} \tag{3}$$

It is well known that any linear program can be rewritten in standard form [1]. The information of $\mathbf{v}_{LB}^k$ and $\mathbf{v}_{UB}^k$ is now in the right-hand side vector $\boldsymbol{\beta}$. Then, for each species k, let $\mathbf{b}^k : D_x \to \mathbb{R}^{n_m^k}$. Harwood and coworkers [2] use lexicographic optimization to render unique exchange fluxes by making them objective function values of a priority list of linear programs. Let $\mathbf{h}^k : D_x \to \mathbb{R}^{n_h^k}$, then:

$$\begin{aligned} \dot{\mathbf{x}}(t) &= \mathbf{f}(t, \mathbf{x}(t), \mathbf{h}^1(\mathbf{x}(t)), \ldots, \mathbf{h}^{n_s}(\mathbf{x}(t))), \ \forall t \in (t_0, t_f], \\ \mathbf{x}(t_0) &= \mathbf{x}_0. \end{aligned} \tag{4}$$

The function $\mathbf{h}^k = [h_1^k \ldots h_{n_h^k}^k]^{\mathrm{T}}$ depends on the solution of a lexicographic linear program:

$$\begin{aligned} h_1^k(\mathbf{x}(t)) = \min_{\mathbf{v} \in \mathbb{R}^{n_v^k}} \ & (\mathbf{c}_1^k)^{\mathrm{T}} \mathbf{v}, \\ \text{s.t. } & \mathbf{A}^k \mathbf{v} = \mathbf{b}^k(\mathbf{x}(t)), \\ & \mathbf{v} \geq \mathbf{0}, \end{aligned} \tag{5}$$

and for $2 \leq i \leq n_h^k$

$$\begin{aligned} h_i^k(\mathbf{x}(t)) = \min_{\mathbf{v} \in \mathbb{R}^{n_v^k}} \ & (\mathbf{c}_i^k)^{\mathrm{T}} \mathbf{v}, \\ \text{s.t. } & \begin{bmatrix} \mathbf{A}^k \\ (\mathbf{c}_1^k)^{\mathrm{T}} \\ \vdots \\ (\mathbf{c}_{i-1}^k)^{\mathrm{T}} \end{bmatrix} \mathbf{v} = \begin{bmatrix} \mathbf{b}^k(\mathbf{x}(t)) \\ h_1^k(\mathbf{x}(t)) \\ \vdots \\ h_{i-1}^k(\mathbf{x}(t)) \end{bmatrix}, \\ & \mathbf{v} \geq \mathbf{0}, \end{aligned} \tag{6}$$

where $\mathbf{c}_i^k \in \mathbb{R}^{n_v^k}$ for $i = 1, \ldots, n_h^k$. A more compact version of (5) and (6) can be obtained by defining the lexicographic minimization operator minL. Let the columns of $\mathbf{C}^k \in \mathbb{R}^{n_v^k \times n_h^k}$ be the vectors $\mathbf{c}_i^k$ for $i = 1, \ldots, n_h^k$. Then,

$$\begin{aligned} \mathbf{h}^k(\mathbf{x}(t)) = \operatorname*{minL}_{\mathbf{v} \in \mathbb{R}^{n_v^k}} \ & (\mathbf{C}^k)^{\mathrm{T}} \mathbf{v}, \\ \text{s.t. } & \mathbf{A}^k \mathbf{v} = \mathbf{b}^k(\mathbf{x}(t)), \\ & \mathbf{v} \geq \mathbf{0}. \end{aligned} \tag{7}$$

Harwood *et al.* [2] present an efficient algorithm to compute a basis that contains optimal bases for all LPs in the priority list. This algorithm was not implemented

in DFBAlab because of difficulties extracting the optimal basis information with no artificial variables from LP solvers in MATLAB, but will be implemented in future releases.

LP Feasibility Problem

A major problem for DFBA simulators is that the LP in (5) may become infeasible as time progresses. In this paper we use the LP feasibility problem [1] combined with lexicographic optimization to generate an extended dynamic system for which the LP always has a solution. An LP feasibility problem finds a feasible point or identifies an LP as infeasible. It has two main characteristics: it is always feasible and its optimal objective function value is zero if and only if the original LP is feasible. Any LP in standard form (3) can be transformed such that $\boldsymbol{\beta} \geq \mathbf{0}$ by multiplying some equality constraints by -1. Then, the LP feasibility problem will have the following general structure [1]:

$$
\begin{aligned}
\min_{\mathbf{v}\in\mathbb{R}^{n_v^k},\, \mathbf{s}\in\mathbb{R}^{n_m^k}} \quad & \sum_{i=1}^{n_m^k} s_i, \\
\text{s.t.} \quad & \mathbf{A}^k\mathbf{v} + \mathbf{s} = \boldsymbol{\beta}, \\
& \mathbf{v} \geq \mathbf{0},\ \mathbf{s} \geq \mathbf{0}.
\end{aligned}
\tag{8}
$$

When an LP is constructed in this form, a feasible solution is obtained by setting $\mathbf{s} = \boldsymbol{\beta}$ and $\mathbf{v} = \mathbf{0}$. DFBAlab uses the LP feasibility problem as the highest priority LP in the lexicographic linear program presented in (5) and (6). Then, the second-priority linear program maximizes biomass and the subsequent lower-priority LPs obtain unique exchange fluxes.

Author details

Process Systems Engineering Laboratory, Massachusetts Institute of Technology, Cambridge, MA 02139.

References

1. Bertsimas, D., Tsitsiklis, J.N.: Introduction to Linear Optimization. Athena Scientific, Belmont, MA (1997)
2. Harwood, S.M., Höffner, K., Barton, P.I.: Solution of ordinary differential equations with a linear program embedded: the right-hand side case. Submitted
